# Routine versus Targeted Viral Load Strategy among Patients Starting Antiretroviral in Hanoi, Vietnam

**DOI:** 10.1002/jia2.25258

**Published:** 2019-03-21

**Authors:** Todd M Pollack, Hao T Duong, Thuy T Pham, Thang D Nguyen, Howard Libman, Long Ngo, James H McMahon, Julian H Elliott, Cuong D Do, Donn J Colby

**Affiliations:** ^1^ The Partnership for Health Advancement in Vietnam (HAIVN) Hanoi Vietnam; ^2^ Department of Medicine Beth Israel Deaconess Medical Center (BIDMC) Boston MA USA; ^3^ Department of Infectious Diseases Bach Mai Hospital (BMH) Hanoi Vietnam; ^4^ Department of Infectious Diseases The Alfred Hospital and Monash University Melbourne Vic. Australia; ^5^ Center for Applied Research on Men and Community Health (CARMAH) Ho Chi Minh City Vietnam; ^6^ SEARCH Thai Red Cross AIDS Research Centre Bangkok Thailand

**Keywords:** HIV, ART, viral load, monitoring, randomized controlled trial, Vietnam

## Abstract

**Introduction:**

HIV viral load (VL) testing is recommended by the WHO as the preferred method for monitoring patients on antiretroviral therapy (ART). However, evidence that routine VL (RVL) monitoring improves clinical outcomes is lacking.

**Methods:**

We conducted a prospective, randomized controlled trial of RVL monitoring every six months versus a targeted VL (TVL) strategy (routine CD4 plus VL testing if clinical or immunological failure) in patients starting ART between April 2011 and April 2014 at Bach Mai Hospital in Hanoi. Six hundred and forty‐seven subjects were randomized to RVL (n = 305) or TVL monitoring (n = 342) and followed up for three years. Primary endpoints were death or WHO clinical Stage 4 events between six and thirty‐six months of ART and rate of virological suppression at three years.

**Results:**

Overall, 37.1% of subjects were female, median age was 33.4 years (IQR: 29.5 to 38.6), and 47% had a CD4 count ≤100 cells/mm^3^ at time of ART initiation. Approximately 44% of study events (death, LTFU, withdrawal, or Stage 4 event) and 68% of deaths occurred within the first six months of ART. Among patients on ART at six months, death or Stage 4 event occurred in 3.6% of RVL and 3.9% of TVL (*p* = 0.823). Survival analysis showed no significant difference between the groups (*p* = 0.825). Viral suppression at 36 months of ART was 97.2% in RVL and 98.9% in TVL (*p* = 0.206) at a threshold of 400 copies/mL and was 98.0% in RVL and 98.9% in TVL (*p* = 0.488) at 1000 copies/mL. In ITT analysis, 20.7% in RVL and 21.9% in TVL (*p* = 0.693) were unsuppressed at 1000 copies/mL.

**Conclusions:**

We found no significant difference in rates of death or Stage 4 events and virological failure in patients with RVL monitoring compared to those monitored with a TVL strategy after three years of follow‐up. Viral suppression rates were high overall and there were few study events among patients alive and on ART after six months, limiting the study's power to detect a difference among study arms. Nonetheless, these data suggest that the choice of VL monitoring strategy may have less impact on patient outcomes compared to efforts to reduce early mortality and improve ART retention.

## 1 Introduction

Routine HIV viral load (RVL) testing is recommended by WHO as the preferred approach for patients on antiretroviral therapy (ART) in low‐ and middle‐income countries (LMIC) 1. Compared to clinical or immunological monitoring, viral load (VL) monitoring has higher sensitivity and positive predictive value for the diagnosis of treatment failure 2, 3, 4, 5. A targeted viral load (TVL) monitoring strategy, where VL testing is used to confirm suspected treatment failure based on clinical and/or immunological criteria, can reduce inappropriate switching to second‐line ART compared to immunological or clinical monitoring alone. However, as with clinical and immunological monitoring, TVL can delay treatment failure diagnosis and second‐line switching and thereby increase the risk of disease progression, selection of antiretroviral drug resistance and HIV transmission 6. RVL monitoring therefore enables more accurate and earlier detection of virological failure, allowing patients to switch to a second‐line regimen at higher CD4 cell counts and before the accumulation of drug resistance mutations 7, 8.

The extent to which RVL monitoring improves clinical outcomes and reduces mortality is less certain. Some observational and modelling studies have suggested that VL monitoring may reduce opportunistic infections (OIs) and improve survival 9, 10, 11. However, three randomized clinical trials, conducted in Uganda, Zambia and Thailand, have failed to demonstrate a difference in clinical outcomes with the addition of VL monitoring to immunological and/or clinical monitoring 12, 13, 14, 15. A 2012 systematic review commissioned by WHO concluded that there was no difference in mortality or new AIDS‐defining illnesses between clinical plus immunological plus virological monitoring and clinical plus immunological monitoring. The authors stated that further research was needed to create a stronger evidence base for HIV monitoring guidelines 16.

Prior to 2016, Vietnam's National HIV Guidelines recommended a TVL strategy with CD4 monitoring every six months and VL testing only when there were signs of clinical or immunological failure 17, 18, 19. To generate data to inform the use of RVL monitoring in Vietnam, we conducted a prospective, randomized controlled trial of RVL monitoring versus TVL monitoring in a patient population starting ART in Vietnam. We hypothesized that RVL monitoring of patients on first‐line ART in Vietnam would result in significantly higher rates of virological suppression and decrease the incidence of death or new or recurrent AIDS‐defining illnesses within three years.

## 2 Methods

### 2.1 Study design and population

The Virologic Monitoring in Viet Nam (VMVN) study was a prospective, randomized controlled trial of RVL monitoring versus TVL monitoring in a patient population starting ART between April 2011 and April 2014 at the outpatient clinic (OPC) of Bach Mai Hospital, a national level hospital in Hanoi, Vietnam. Participants were patients registered at the OPC who met the following inclusion criteria: (1) age ≥18 years old; (2) confirmed HIV infection; (3) eligible for ART according to criteria defined by Vietnam Ministry of Health (MOH) HIV guidelines 17, 18; and (4) ART‐naïve or off ART for at least three months prior to enrolment. Patients with a history of treatment failure or known resistance to first‐line ART were excluded.

The sample size was calculated with a two‐sided alpha of 0.05 and an 80% power to detect a 40% relative reduction in the primary outcome of death or new clinical Stage 4 event from six to thirty‐six months after ART initiation. At the time of the study protocol development, it was reported that up to 28% of patients on ART in Hanoi did not achieve viral suppression 20 and that mortality in the Vietnam national ART programme was 17.2% over 36 months, of which half occurred after six months of treatment 21. Based on these data, it was estimated that the primary outcome would occur in 24% of subjects in the control (TVL) arm. A 10% absolute reduction, or 40% relative reduction, in the primary outcome to 14% in the RVL arm was considered to be both clinically significant and a threshold that could potentially influence public policy. The calculated sample size of 578 was increased to a total of 650 to account for a dropout and lost to follow‐up proportion of 10%.

HIV‐positive patients who presented to the OPC for care within a common time frame (e.g. same month) were grouped together and initiated on ART at the same time for the convenience of counselling and follow‐up. Each treatment group included between 10 and 20 patients. Consecutive groups of eligible patients were randomly assigned to either the control (TVL) or intervention (RVL) group. As the size of the groups varied, the total number of patients was not the same between the two arms. In total, 657 patients signed the consent form, but nine were excluded for not meeting all eligibility requirements. The remaining sample (n = 648) was randomized into the two groups. One patient in the control group died before ART initiation and was excluded.

Clinical management of patients in the study followed the Vietnam MOH HIV guidelines and the OPC's standard practice. The recommended first‐line ART regimen was zidovudine, lamivudine and nevirapine for patients initiating ART in 2011 and 2012 17, and was tenofovir, lamivudine and efavirenz for those initiating ART after 2012 18, 19. Patients were seen in the OPC for clinical follow‐up either monthly or every two months according to the discretion of the treating physician. Clinic protocol was monthly follow‐up after initiation of ART and every two‐month follow‐up once the patient had been on ART for at least six months and was clinically stable with good adherence. The presence or absence of a VL test was not a criteria for determining the interval of follow‐up. Study subjects received a quarterly study clinical exam with clinical screening for OIs. All subjects received CD4 count testing every six months. In the TVL arm, VL was performed only when the patient met clinical and/or immunological criteria for treatment failure per the Vietnam MOH guidelines 18. Patients in the RVL arm received a VL every six months in addition to the CD4 cell count. Patients with a VL ≥1000 copies/mL received monthly adherence counselling sessions and a repeat VL after three months. If the repeat VL remained above 1000 copies/mL or if a genotype test showed drug resistance mutations, then the patient was considered for second‐line ART. Genotype testing was not included in the Vietnam public ART programme at the time of the study implementation, but could be conducted if ordered by a clinic physician and paid for by the patient's own resources. Second‐line ART regimens were tenofovir or zidovudine combined with lamivudine and lopinavir/ritonavir. At the end of three years of follow‐up, patients in both arms had a VL test to determine the rate of virological suppression.

### 2.2 Ethical considerations

The study was approved by the Institutional Review Board of Beth Israel Deaconess Medical Center (#2010P000334) in Boston, USA and the Ethical Committee of Bach Mai Hospital in Hanoi, Vietnam. All subjects provided written informed consent prior to study participation. The VMVN study was registered at www.clinicaltrials.gov (ClinicalTrials.gov identification number: NCT01317498). This study conforms to the ethical standards for human subjects research of the Declaration of Helsinki.

### 2.3 Variables, measures and definitions

Data collected at baseline included gender, age, number of years of education, body mass index (BMI), HIV transmission route, history of tuberculosis (TB) and current TB treatment, history of OIs and current OIs, WHO clinical stage, CD4 cell count, prior ART history and baseline ART regimen.

Weight status was categorized as underweight (BMI < 18.5), healthy weight (18.5 ≤ BMI < 25), overweight or obese (BMI ≥ 25). HIV transmission route was categorized as injection drug use with or without other route, heterosexual transmission only, and other. Current OIs were recorded by the study doctor during the clinical examination at enrolment.

Plasma VL was measured using the COBAS^®^ AmpliPrep/COBAS^®^ TaqMan^®^ HIV‐1 Test v2.0 kit (Roche Molecular Systems, Branchburg, NJ).

### 2.4 Study outcomes

The primary outcomes of the study were death or new/recurrent clinical Stage 4 conditions between six and thirty‐six‐months of ART, and VL suppression at the 36‐month visit. Deaths were determined as AIDS‐related or non‐AIDS related by the study doctor. Determination of a clinical Stage 4 condition was based on review by an independent committee of experienced HIV physicians following the WHO diagnostic criteria for OIs 22. To minimize potential bias, the committee was blinded to study grouping. VL results were not used to make the determination of new or recurrent Stage 4 condition. Only those events determined by the committee to have sufficient evidence for a clinical or definitive diagnosis of a new/recurrent Stage 4 event were considered to have met the criteria for a study outcome.

### 2.5 Data analysis

Distributions of selected demographic characteristics and baseline clinical variables between the RVL group and the TVL group were presented and compared using either chi‐square or Fisher's exact test for categorical variables, and two‐sample *t*‐test or nonparametric equality‐of‐medians test for continuous variables.

#### 2.5.1 Outcome 1: time to death or new/recurrent clinical Stage 4 condition

The survival time for a non‐censored subject was the number of days from the ART start date to either the date of death or the date of a new/recurrent clinical Stage 4 diagnosis. A subject was censored if the subject dropped out of the study (e.g. transfer out, lost to follow‐up (LTFU), study withdrawal, or discontinuation of ART) or if the subject was followed until 36 months and did not have a study event. LTFU was defined as no clinic visit for at least three consecutive months. Subjects switched to second‐line ART without a clinical Stage 4 event were not censored and were included in the analysis as a non‐event. Survival time for a censored subject was the number of days from the ART start date to the date of last clinic visit. Due to the small number of subjects with non‐AIDS‐related deaths, competing risks analysis was not performed.

As the monitoring of the study groups differed only from the time of the first RVL at six months, only patients who received ART for at least six months were included in the survival analysis. Deaths or other events (e.g. LTFU, transfer out, study withdraw, or drop out for other reasons) at or before six months of ART were excluded. Patients with a clinical Stage 4 condition occurring at or before six months of ART were included in the survival analysis but their Stage 4 event was not counted.

The product‐limit Kaplan–Meier estimates were used to construct the survival functions for the two study groups. The log‐rank test was used to test for the difference in these two survival functions. The proportional hazard assumption was tested using Schoenfeld residuals.

#### 2.5.2 Outcome 2: proportion of patients with VL <400 copies/mL at 36 months

Fisher's exact test was used to compare the proportion of patients with VL <400 copies/mL at 36 months between the two groups. We also calculated and compared the proportion of patients with VL <1000 at 36 months between the two groups. In addition, an intention‐to‐treat (ITT) analysis was used in which those who did not have a VL at 36 months due to death or other study event were considered as having a VL at or above the cutoff point.

All analyses were performed using Stata/SE 14.0 (Stata Corporation, College Station, TX).

## 3 Results

### 3.1 Characteristics of the study participants

**Figure 1:**
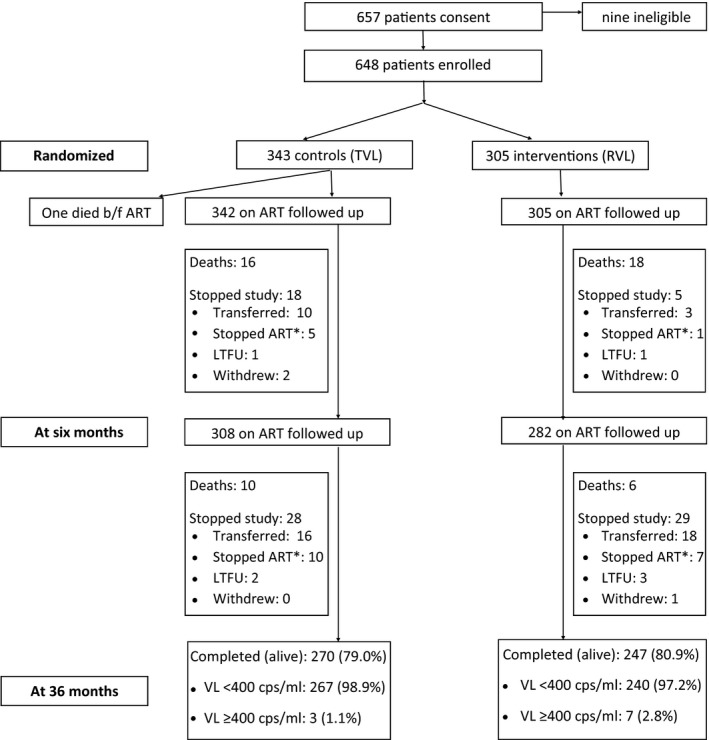
Study overview. *Due to entry to jail or drug rehabilitation.

A total of 647 subjects were randomized to either RVL monitoring (n = 305) or TVL monitoring (n = 342) (Figure 1). Overall, 37.1% of the subjects were female, the median age was 33.4 years (IQR: 29.5 to 38.6) and the median baseline CD4 cell count was 130 cells/mL (IQR: 32 to 287). Table 1 presents the demographics and baseline clinical characteristics.

**Table 1:** Demographics and baseline clinical data by the treatment group

	RVL N (%)	TVL N (%)	*p*‐value
Total	305 (47.1%)	342 (52.9%)	** **
Gender			
Male	190 (62.3%)	217 (63.4%)	0.761^a^
Female	115 (37.7%)	125 (36.6%)	
Age			
N, mean ± SD	305, 34.9 ± 8.0	342, 35.2 ± 9.3	0.622^c^
N, median (range)	305, 33.4 (18.5 to 63.1)	342, 33.6 (20.1 to 74.1)	0.553^d^
<25	19 (6.2%)	33 (9.7%)	0.230^a^
25 to <30	60 (19.7%)	63 (18.4%)	
30 to<35	111 (36.4%)	106 (31.0%)	
35+	115 (37.7%)	140 (40.9%)	
Years of education			
≤5	22 (7.2%)	20 (5.8%)	0.169^b^
6 to 9	90 (29.5%)	119 (34.8%)	
10 to 12	124 (40.7%)	148 (43.3%)	
>12	64 (21.0%)	53 (15.5%)	
Missing	5 (1.6%)	2 (0.6%)	
BMI			
<18.5	104 (34.1%)	117 (34.2%)	0.966^a^
≥18.5 but <25	188 (61.6%)	209 (61.1%)	
≥25	13 (4.3%)	16 (4.7%)	
Transmission route of HIV			
Heterosexual	218 (71.5%)	217 (63.4%)	0.094^a^
IV drug use	77 (25.2%)	110 (32.2%)	
Other	10 (3.3%)	15 (4.4%)	
Prior TB diagnosis			
Yes	36 (11.8%)	58 (17.0%)	0.063^a^
No	269 (88.2%)	284 (83.0%)	
TB treatment status			
Currently under TB treatment	29 (9.5%)	43 (12.6%)	0.237^b^
Completed treatment	3 (1.0%)	9 (2.6%)	
Unknown	4 (1.3%)	6 (1.8%)	
N/A	269 (88.2%)	284 (83.0%)	
Ever diagnosed with an OI			
Yes	130 (42.6%)	130 (38.0%)	0.232^a^
No	175 (57.4%)	212 (62.0%)	
Current OI manifestation			
Yes	148 (48.5%)	153 (44.7%)	0.335^a^
No	157 (51.5%)	189 (55.3%)	
Clinical stage at enrolment			
I	134 (44.0%)	166 (48.5%)	0.415^a^
II	26 (8.5%)	22 (6.4%)	
III	37 (12.1%)	32 (9.4%)	
IV	108 (35.4%)	122 (35.7%)	
CD4 at enrollment			
≤100	140 (45.9%)	161 (47.1%)	0.490^a^
101 to 250	73 (23.9%)	69 (20.2%)	
>250	92 (30.2%)	112 (32.7%)	
Prior ARV treatment			
Yes	10 (3.3%)	16 (4.7%)	0.366^a^
No	295 (96.7%)	326 (95.3%)	
Baseline ART regimen			
d4T + 3TC + NVP	1 (0.3%)	3 (0.9%)	0.439^b^
d4T + 3TC + EFV	0	5 (1.4%)	
AZT + 3TC + NVP	90 (29.5%)	96 (28.1%)	
AZT + 3TC + EFV	36 (11.8%)	45 (13.2%)	
TDF + 3TC + NVP	4 (1.3%)	6 (1.7%)	
TDF + 3TC + EFV	172 (56.4%)	185 (54.1%)	
Other	2 (0.7%)	2 (0.6%)	

^a^Chi‐square test. ^b^Fisher's exact test. ^c^Two‐sample *t*‐test. ^d^Nonparametric equality‐of‐medians test.

#### 3.1.1 Outcome 1: death or new/recurrent clinical Stage 4 condition

Fifty (7.7%) patients died during the study; 24 in the RVL arm and 26 in the TVL arm. Seven of the deaths were considered to be non‐AIDS related. Thirteen patients had 14 new or recurrent clinical Stage 4 events. One patient had two Stage 4 events; one occurring before and one occurring after six months of ART. Two of the 13 patients with a new or recurrent Stage 4 condition died afterward. In addition, 80 patients did not complete the study; 13.4% (n = 46) in the TVL group and 11.2% (n = 34) in the RVL group respectively (*p* = 0.374). Reasons for not completing the study included transfer out (58.7%), LTFU (8.8%), study withdrawal (3.8%), and discontinuation of ART due to entry to jail or drug rehabilitation (28.7%). Of the 61 all‐cause deaths or new/recurrent clinical Stage 4 events included in this analysis, 22 (36.1%) (sixteen all‐cause deaths and six new/recurrent clinical Stage 4 events) occurred after six months of ART. Of these, twelve occurred in the TVL group (ten deaths, two clinical Stage 4 events) and ten in the RVL group (six deaths, four clinical Stage 4 events) (*p* = 0.823) (Table 2).

**Table 2:** Proportion of patients alive and on ART at six months with study events, by treatment group, (n = 590)

Study group	All deaths or new/recurrent Stage 4 event		HIV/AIDS‐related deaths or new/recurrent Stage 4 event^a^	
	Patients with event	Patients without event	Patients with event	Patients without event
RVL	10 (3.6% [1.9 to 6.5])	272 (96.4% [93.5 to 98.1])	8 (2.9% [1.4 to 5.6])	272 (97.1% [94.4 to 98.6])
TVL	12 (3.9% [2.2 to 6.8])	296 (96.1% [93.2 to 97.8])	10 (3.3% [1.8 to 6.0])	296 (96.7% [94.0 to 98.2])
*p*‐value	0.823		0.773	

RVL, routine VL; TVL, targeted VL.

a Excludes patients with non‐HIV/AIDS‐related deaths.

Fifty‐seven patients were excluded from the survival analysis including 34 patients who died and 23 patients who left the study at or before six months of ART. Five patients who had a clinical Stage 4 event at or before six months of ART were included in the analysis but their Stage 4 event was not counted.

**Figure 2:**
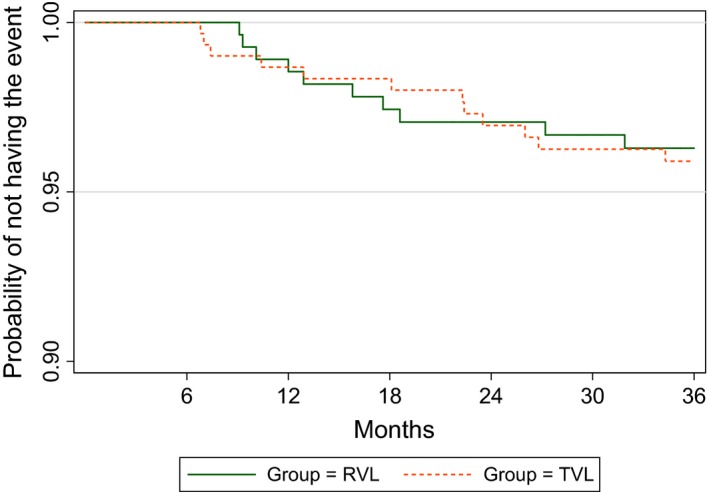
All deaths or new/recurrent Stage 4 events – Kaplan–Meier survival curve.

Figure 2 shows the product‐limit Kaplan–Meier estimate of the survival function. This analysis included all‐cause deaths (n = 16) and new/recurrent clinical Stage 4 events (n = 6). The same analysis was carried out but with the exclusion of the four subjects whose deaths were non‐AIDS related (n = 18). The survival analysis in both cases shows no statistically significant difference in survival functions between the two groups (*p* = 0.825, and *p* = 0.775 respectively).

#### 3.1.2 Outcome 2: proportion of patients with VL <400 copies/mL at 36 months of ART

Table 3 shows the proportion of patients with VL suppression at 36 months of ART at different VL thresholds. There were 517 patients who completed their 36‐month visit (247 (80.9%) in the RVL and 270 (79.0%) in the TVL group). Viral suppression at a threshold of 400 copies/mL was 97.2% in the RVL group and 98.9% in the TVL group at 36 months (*p* = 0.206). Using a threshold of 1000 copies/mL, viral suppression was 98.0% in the RVL group and 98.9% in the TVL group (*p* = 0.488).

**Table 3:** Proportion of patients with viral suppression at 36 months between the two groups (n = 517)

Cutoff	RVL N (%, 95%CI)	TVL N (%, 95%CI)	*p*‐value
	247 (100%)	270 (100%)	
1000 copies/mL			
<1000 copies/mL	242 (98.0% [95.2 to 99.2])	267 (98.9% [96.6 to 99.6])	0.488^a^
≥1000 copies/mL	5 (2.0% [0.8 to 4.8])	3 (1.1% [0.4 to 3.4])	
400 copies/mL			
<400 copies/mL	240 (97.2% [94.1 to 98.7])	267 (98.9% [96.6 to 99.6])	0.206^a^
≥400 copies/mL	7 (2.8% [1.3 to 5.9])	3 (1.1% [0.4 to 3.4])	

RVL, routine VL; TVL, targeted VL

a Fisher's exact test.

Results from the ITT analysis are presented in Table 4. There was no significant difference in the VL suppression rate between the two groups at either cutoff point.

**Table 4:** Intention to Treat Analysis: Proportion of patients with viral suppression at 36 months between the two groups (n = 647)

Cutoff	RVL N (%, 95% CI)	TVL N (%, 95% CI)	*p*‐value
	305 (100%)	342 (100%)	
1000 copies/mL			
<1000 copies/mL	242 (79.3% [74.4 to 83.5])	267 (78.1% [73.3 to 82.2])	0.693
≥1000 copies/mL	63 (20.7% [16.5 to 25.6])	75 (21.9% [17.8 to 26.7)	
400 copies/mL			
<400 copies/mL	240 (78.7% [73.7 to 82.9])	267 (78.1% [73.3 to 82.2])	0.849
≥400 copies/mL	65 (21.3% [17.1 to 26.3])	75 (21.9% [17.8 to 26.7])	

RVL, routine VL; TVL, targeted VL.

Twenty‐nine patients (9.5%) in the RVL arm and nine patients (2.6%) in the TVL arm had at least one VL >1000 copies/mL at any point during the 36 months of follow‐up. Overall, 15 patients switched to a second‐line ART regimen due to virological treatment failure; nine in the RVL arm and six in the TVL arm (*p* = 0.313). The median time from treatment initiation to second‐line switch was 13.3 months in the RVL arm and 14.9 months in the TVL arm (*p* = 0.833). Of the 15 patients who switched to second‐line ART due to treatment failure, 13 completed the study follow‐up period and 12 of the 13 had a VL <400 copies/mL at the 36‐month visit. Among the 20 patients in the RVL arm with high VL that did not switch to second‐line, twelve had a repeat VL <1000 copies/mL, one died, one stopped ART, one transferred out, and five had their high VL result at the final 36‐month study visit. Among the three patients in the TVL arm with high VL that did not switch to second‐line, one had a repeat VL <1000 copies/mL, and two had their high VL result at the final 36‐month study visit. Retrospective testing of stored plasma samples from five of the nine patients in the TVL group with VL >1000 copies/mL during the study showed that RVL testing would have led to earlier detection of virological failure in four of the five cases (range: six to thirty months).

## 4 Discussion

We conducted a prospective, randomized controlled trial to evaluate the effect of RVL monitoring on clinical outcomes and survival among HIV‐infected patients initiating ART at one of the largest HIV clinics in Vietnam. After 36 months of follow‐up, we found no difference in mortality and rates of new or recurrent clinical stage 4 conditions compared to patients monitored with a TVL strategy. Viral suppression rates at 36 months of ART were greater than 97% and there was no difference between the two study arms.

The WHO recommends RVL as the preferred strategy for monitoring patients on ART, with TVL as an alternative in settings with limited access to VL testing, although the recommendation is based on low quality evidence 1. Many LMICs are currently in the process of scaling up of RVL despite technical, logistical and financial challenges 23, 24. Our study was intended to generate data to assist policy makers in Vietnam and other LMICs with the scale‐up of VL testing. Although we did not find a difference between the two monitoring strategies, our results should be interpreted with some caution. Among patients alive and on ART at six months, death or Stage 4 events occurred in only 3.7% of patients and more than 97% of patients had a VL below 400 copies/mL at 36 months. These outcomes are significantly better than those reported in previous studies in Vietnam 25, 26, 27, 28, including reports of 8.1% mortality from six to thirty‐six months on ART nationally 21, and virological non‐suppression of 28% in Hanoi 20. The study's primary outcome, therefore, occurred at a rate much lower than was expected in both study arms, leaving the study underpowered to detect a difference in the primary outcome.

Our findings are consistent with data previously reported in Uganda 13, 15 and Thailand 12. The Ugandan study compared clinical outcomes among patients with one of three monitoring strategies, including a clinical arm (weekly home visits), CD4 arm (quarterly CD4 cell counts and weekly home visits) and VL arm (quarterly CD4 cell count and VL testing and weekly home visits), over a median follow‐up of three years. The study found no significant difference between the CD4 arm and the VL arm in the rate of new AIDS‐defining events or death (adjusted HR = 1.23, 95% CI = 0.82 to 1.84) 15. Following the initial analysis, individuals in the clinical arm were re‐randomized to the other two arms and all participants were followed up for a median follow‐up of 5.2 years 13. Once again, no association was found between the monitoring arms and new AIDS defining events or death (adjusted HR = 1.19 for CD4‐only vs. CD4‐VL; 95% CI 0.82 to 1.73).

The Thai study compared a second‐line switching strategy based on CD4‐only monitoring versus VL monitoring 12. The primary endpoint was clinical failure at three years, defined as death, new AIDS‐defining event, or CD4 count <50 cells/mm^3^. After three years of follow‐up, the cumulative risk of clinical failure was 8.0% (95% CI 5.6 to 11.4) with VL monitoring compared to 7.4% (95% CI 5.1 to 10.7) with CD4‐only monitoring; a difference which met the pre‐determined non‐inferiority criterion. At study completion, there were no differences in viral suppression or immune restoration between the two arms.

While evidence demonstrating mortality benefit of RVL monitoring is lacking, previous studies have shown that RVL monitoring results in earlier detection of virological failure, earlier switching to second‐line therapy, reduced duration of uncontrolled viraemia, and less drug resistance at the time of failure compared to TVL 7, 8, 29. In our study, we found that RVL testing would have resulted in earlier detection of virological failure in 80% (4/5) of patients in the TVL arm who had VL >1000 copies/mL during the study period (retrospective testing of plasma VL was not available for four patients). We also found that a greater proportion of patients with VL >1000 copies/mL in the TVL arm (6/9) switched to second‐line compared to those in the RVL arm (9/29). In those that did switch, there was no difference between study arms in the median time from treatment initiation to second‐line ART switch. Realizing the benefits of earlier detection depends on the application of appropriate interventions for patients with high VL results. For patients with VL >1000 copies/mL, Vietnam's National HIV guideline recommends monthly adherence counselling sessions and repeat VL testing after three months. Patients should be switched to second‐line ART if the repeat VL, tested while ARVs are taken with good adherence, remain above 1000 copies/mL. Although we did not measure the compliance with or quality of adherence interventions for patients with high VL results, it is possible that earlier detection of virological failure in the RVL arm may have improved the chances of VL resuppression following the counselling intervention. Nonetheless, demonstrating a clinical benefit of earlier detection and intervention may not have been possible within the three‐year follow‐up period of our study.

Bach Mai Hospital is a tertiary level referral centre and is well known for providing a high quality of HIV care. In addition, adherence rates were high among patients in this study. Data collected every three months showed that only 2.2% of the patients were late or missed any follow‐up visits in the last three months, and adherence to ART was recorded as good (taking >95% of doses) in the medical record in 97.8% of the patients. It is possible that a very high adherence rate may have contributed to good patient outcomes, minimizing the benefit of RVL monitoring. Several studies have evaluated the use of virological failure prediction tools using clinical and laboratory risk factors to select clinically stable patients who may be exempted from VL testing at RVL monitoring milestones 30, 31. This approach to RVL monitoring may be more cost‐effective in populations with low rates of treatment failure 32.

The majority of clinical events in this study occurred before six‐months of ART and before the first VL was indicated. Late presentation to care is a well‐known risk factor for mortality. In our study, 66.3% of patients met criteria for advanced HIV disease (which is defined by the WHO as CD4 cell count <200 cells/mm^3^ or a WHO clinical Stage 3 or 4 event) at the time of ART initiation. People with advanced HIV disease are at high risk of death, even after starting ART 33. Additionally, despite the high viral suppression rate, more than 20% of patients failed using an ITT analysis. This suggests that earlier HIV diagnosis, use of enhanced OI prophylaxis, rapid initiation of ART, and efforts to reduce LTFU and improve ART retention may have more impact in patient outcomes than the particular approach to ART monitoring 34, 35, 36, 37.

It is possible that a different VL testing schedule could lead to different results. A study from South Africa concluded that VL testing done at three months post‐ART initiation is associated with better outcomes than VL testing performed at six months 38. A VL at three months may allow for earlier detection of adherence problems. This may be particularly relevant as integrase inhibitors are increasingly being used in first‐line regimens in LMIC and result in more rapid suppression of VL when compared to Efavirenz‐based regimens 39, 40.

As discussed above, the major limitation of our study is that it was underpowered to detect a difference in the primary outcome between the two arms. At the time that the study was conceived, all first‐line ART regimens in Vietnam contained one or more of the ARV drugs d4T, AZT and NVP; all of which have high rates of toxicities and side effects. All three drugs were removed from the recommended first‐line regimen in Vietnam before the study ended. In addition, the number of patients on ART in the country more than doubled over the same time period. It may be that better tolerated ARV drugs, more experienced healthcare workers, and more resources devoted to HIV in the healthcare system led to better treatment outcomes, thus lowering the power of the study to detect a difference in clinical outcomes between the two study arms.

Our study has several other limitations. First, study participants were randomized by ART group rather than by individual. It is possible that the group randomization could have introduced bias. To reduce the risk of selection bias, clinic staff were blinded to the group assignments until after patients were assigned and ART was initiated. Notably, participant demographic and baseline clinical characteristics did not differ between the study arms. Second, we did not collect data on the interval of patient follow‐up (e.g. monthly or every two months). It is possible that a difference in follow‐up interval could have introduced bias. As a high VL result would have triggered more frequent follow‐up, it is possible that monthly follow‐up was more common in the RVL group. If that was the case, it could potentially have biased the results toward better outcomes for the RVL group. Third, our follow‐up period was three‐years following ART initiation. It is possible that a longer follow‐up period may be needed to demonstrate the benefits of RVL monitoring. However, Okoboi followed their patents a median of 5.2 years and had similar findings to our study 13. Fourth, genotype resistance testing was not performed for all patients with virological failure. As a result, we could not assess the impact of RVL on HIV drug resistance at the time of failure. Fifth, our study was conducted at a single hospital‐based OPC in Hanoi, considered to be a centre of excellence for HIV care in the country. The results, therefore, cannot be generalized to other clinical settings, such as those located in rural or mountainous areas of Vietnam where patient adherence may be worse and clinical outcomes more common. Finally, although unlikely, we cannot rule out the possibility that patients in the TVL group accessed RVL testing outside the study setting.

## 5 Conclusions

In this study population, there was no significant difference in the rates of death, new/recurrent clinical Stage 4 events, or viral suppression in patients monitored with RVL every six months compared to those monitored with a TVL strategy after three years of follow‐up. In our highly adherent patients managed at a national level HIV clinic, there were high rates of viral suppression and relatively few study outcomes among patients alive and on ART after six months. As a result, the study was likely underpowered to detect a difference in the primary outcomes between the two arms. The WHO recommends RVL for accurate and early diagnosis of treatment failure. Nonetheless, our data suggest that the choice of VL monitoring strategy may have less impact on patient outcomes compared to efforts to reduce early mortality and improve ART retention.

## Competing interest

All authors have no conflicts of interests.

## Authors’ contributions

All authors have read and approved the final manuscript. DJC, TTP, JHM, JHE and HL designed the research study. TMP, DJC, TDN, TTP and CDD performed the research. LN and HD analysed the data. TP and HD wrote the paper.

## References

[1] WHO . Consolidated guidelines on the use of antiretroviral drugs for treating and preventing HIV infection – recommendations for a public health approach. 2016. 27466667

[2] Reynolds SJ , Nakigozi G , Newell K , Ndyanabo A , Galiwongo R , Boaz I , et al. Failure of immunologic criteria to appropriately identify antiretroviral treatment failure in Uganda. AIDS. 2009;23:697–700. 19209067 10.1097/QAD.0b013e3283262a78 PMC2720562

[3] Moore DM , Awor A , Downing R , Kaplan J , Montaner JSG , Hancock J , et al. CD4+ T‐cell count monitoring does not accurately identify HIV‐infected adults with virologic failure receiving antiretroviral therapy. J Acquir Immune Defic Syndr. 2008;49:477–84. 18989232 10.1097/QAI.0b013e318186eb18

[4] Kantor R , Diero L , Delong A , Kamle L , Muyonga S , Mambo F , et al. Misclassification of first‐line antiretroviral treatment failure based on immunological monitoring of HIV infection in resource‐limited settings. Clin Infect Dis. 2009;49:454–62. 19569972 10.1086/600396 PMC4859427

[5] Mee P , Fielding KL , Charalambous S , Churchyard GJ , Grant AD . Evaluation of the WHO criteria for antiretroviral treatment failure among adults in South Africa. AIDS. 2008;22:1971–7. 18784460 10.1097/QAD.0b013e32830e4cd8

[6] WHO . Consolidated guidelines on the use of antiretroviral drugs for treating and preventing HIV infection – recommendations for a public health approach. 2013. 24716260

[7] Sungkanuparph S , Manosuthi W , Kiertiburanakul S , Piyavong B , Chumpathat N , Chantratita W . Options for a second‐line antiretroviral regimen for HIV type 1‐infected patients whose initial regimen of a fixed‐dose combination of stavudine, lamivudine, and nevirapine fails. Clin Infect Dis. 2007;44:447–52. 17205457 10.1086/510745

[8] Calmy A , Ford N , Hirschel B , Reynolds SJ , Lynen L , Goemaere E , et al. HIV viral load monitoring in resource‐limited regions: optional or necessary? Clin Infect Dis. 2007;44:128–34. 17143828 10.1086/510073

[9] Kimmel AD , Weinstein MC , Anglaret X , Goldie SJ , Losina E , Yazdanpanah Y , et al. Laboratory monitoring to guide switching antiretroviral therapy in resource‐limited settings: Clinical benefits and cost‐effectiveness. J Acquir Immune Defic Syndr. 2010;54:258–68. 20404739 10.1097/QAI.0b013e3181d0db97 PMC3174771

[10] Keiser O , Chi BH , Gsponer T , Boulle A , Orrell C , Phiri S , et al. Outcomes of antiretroviral treatment in programmes with and without routine viral load monitoring in southern Africa. AIDS. 2011;25:1761–9. 21681057 10.1097/QAD.0b013e328349822f PMC3605707

[11] Estill J , Egger M , Johnson LF , Gsponer T , Wandeler G , Davies MA , et al. Monitoring of antiretroviral therapy and mortality in HIV programmes in Malawi, South Africa and Zambia: mathematical modelling study. PLoS One. 2013;8:e57611. 23469035 10.1371/journal.pone.0057611 PMC3585414

[12] Jourdain G , Le Cœur S , Ngo‐Giang‐Huong N , Traisathit P , Cressey TR , Fregonese F , et al. Switching HIV treatment in adults based on CD4 count versus viral load monitoring: a randomized, non‐inferiority trial in Thailand. PLoS Med. 2013;10:e1001494. 23940461 10.1371/journal.pmed.1001494 PMC3735458

[13] Okoboi S , Ekwaru PJ , Campbell JD , Egessa A , King R , Bakanda C , et al. No differences in clinical outcomes with the addition of viral load testing to CD4 cell count monitoring among HIV infected participants receiving ART in rural Uganda: long‐term results from the Home Based AIDS Care Project Global health. BMC Public Health. 2016;16:101. 26830678 10.1186/s12889-016-2781-y PMC4736157

[14] Saag M , Westfall A , Luhanga D , Mulenga P , Chi B , Mulenga L ., et al. A cluster randomized trial of routine vs discretionary viral load monitoring among adults starting ART: Zambia. 19th Annual Conference on Retroviruses & Opportunistic Infections. 2012.

[15] Mermin J , Ekwaru JP , Were W , Degerman R , Bunnell R , Kaharuza F , et al. Utility of routine viral load, CD4 cell count, and clinical monitoring among adults with HIV receiving antiretroviral therapy in Uganda: randomised trial. BMJ. 2011;343:d6792. 22074711 10.1136/bmj.d6792 PMC3213241

[16] WHO . Strategies for optimizing HIV monitoring among adults, children and pregnant women living with HIV receiving antiretroviral therapy: a systematic review. 2013.

[17] MOH . Guidelines for HIV/AIDS diagnosis, treatment, and care. 2009.

[18] MOH . Guidelines for HIV/AIDS diagnosis, treatment, and care. 2011.

[19] MOH . Guidelines for HIV/AIDS diagnosis, treatment, and care. 2015.

[20] Jordan MR , La H , Nguyen HD , Sheehan H , Lien TT , Duong DV , et al. Correlates of HIV‐1 viral suppression in a cohort of HIV‐positive drug users receiving antiretroviral therapy in Hanoi, Vietnam. Int J STD AIDS. 2009;20(6):418–22. 19451329 10.1258/ijsa.2008.008389 PMC2887676

[21] Nguyen DB , Do NT , Shiraishi RW , Le YN , Tran QH , Huu Nguyen H , et al. Outcomes of antiretroviral therapy in Vietnam: results from a national evaluation. PLoS One. 2013;8:e55750. 23457477 10.1371/journal.pone.0055750 PMC3574016

[22] WHO . Antiretroviral therapy for HIV infection in adults and adolescents: recommendations for a public health approach. 2010. 23741771

[23] Roberts T , Cohn J , Bonner K , Hargreaves S . Scale‐up of routine viral load testing in resource‐poor settings: current and future implementation challenges. Clin Infect Dis. 2016;62:1043–8. 26743094 10.1093/cid/ciw001 PMC4803106

[24] Lecher S , Ellenberger D , Kim AA , Fonjungo PN , Agolory S , Borget MY , et al. Scale‐up of HIV viral load monitoring – seven sub‐Saharan African countries. MMWR Morb Mortal Wkly Rep. 2015;64:1287–90. 26605986 10.15585/mmwr.mm6446a3

[25] Rangarajan S , Donn JC , Giang LT , Bui DD , Hung Nguyen H , Tou PB , et al. Factors associated with HIV viral load suppression on antiretroviral therapy in Vietnam. J Virus Erad. 2016;2:94–101. 27482442 10.1016/S2055-6640(20)30466-0 PMC4965252

[26] Tanuma J , Matsumoto S , Haneuse S , Cuong DD , Vu TV , Thuy PTT , et al. Long‐term viral suppression and immune recovery during first‐line antiretroviral therapy: a study of an HIV‐infected adult cohort in Hanoi, Vietnam. J Int AIDS Soc. 2017;20:e25030. 10.1002/jia2.25030 PMC5810334 29211347

[27] Trinh TT , Montague BT , Flanigan TP , Gerard HM . HIV suppression among patients on treatment in Vietnam: a review of HIV viral load testing in a public urban clinic in Ho Chi Minh City. AIDS Res Treat. 2011;2011:230953. 21490776 10.1155/2011/230953 PMC3066628

[28] Cuong DD , Sönnerborg A , Van Tam V , El‐Khatib Z , Santacatterina M , Marrone G , et al. Impact of peer support on virologic failure in HIV‐infected patients on antiretroviral therapy – a cluster randomized controlled trial in Vietnam. BMC Infect Dis. 2016;16:759. 27986077 10.1186/s12879-016-2017-x PMC5162085

[29] Gupta RK , Hill A , Sawyer AW , Cozzi‐Lepri A , von Wyl V , Yerly S , et al. Virological monitoring and resistance to first‐line highly active antiretroviral therapy in adults infected with HIV‐1 treated under WHO guidelines: a systematic review and meta‐analysis. Lancet Infect Dis. 2009;9:409–17. 19555900 10.1016/S1473-3099(09)70136-7

[30] Phan V , Thai S , Koole O , Menten J , Meheus F , van Griensven F , et al. Validation of a clinical prediction score to target viral load testing in adults with suspected first‐line treatment failure in resource‐constrained settings. J Acquir Immune Defic Syndr. 2013;62:509–16. 23334504 10.1097/QAI.0b013e318285d28c

[31] Griensven JV , Phan V , Thai S , Koole O , Lynen L . Simplified clinical prediction scores to target viral load testing in adults with suspected first line treatment failure in Phnom Penh, Cambodia. PLoS One. 2014;1:e87879. 10.1371/journal.pone.0087879 PMC3913697 24504463

[32] Mungwira RG , Divala TH , Nyirenda OM , Kanjala M , Muwalo F , Mkandawire FA , et al. A targeted approach for routine viral load monitoring in Malawian adults on antiretroviral therapy. Trop Med Int Health. 2018;23(5):526–32. 29505108 10.1111/tmi.13047 PMC5932246

[33] Ford N , Doherty M . The enduring challenge of advanced HIV infection. N Engl J Med. 2017;377:283–4. 28723319 10.1056/NEJMe1707598

[34] Losina E , Touré H , Uhler LM , Anglaret X , Paltiel AD , Balestre E , et al. Cost‐effectiveness of preventing loss to follow‐up in HIV treatment programs: a Côte d'Ivoire appraisal. PLoS Med. 2009;6:e1000173. 19859538 10.1371/journal.pmed.1000173 PMC2762030

[35] McCreesh N , Andrianakis I , Nsubuga RN , Strong M , Vernon I , McKinley TJ , et al. Universal test, treat, and keep: improving ART retention is key in cost‐effective HIV control in Uganda. BMC Infect Dis. 2017;17:322. 28468605 10.1186/s12879-017-2420-y PMC5415795

[36] Post FA , Szubert AJ , Prendergast AJ , Johnston V , Lyall H , Fitzgerald F , et al., PSR of Ea mortaLITY in H adults and children starting antiretroviral therapy (REALITY) TT . Causes and timing of mortality and morbidity among late presenters starting antiretroviral therapy in the REALITY trial. Clin Infect Dis. 2018;66:S132–9. 29514234 10.1093/cid/cix1141 PMC5850430

[37] WHO . Guidelines for managing advanced HIV disease and rapid initiation of antiretroviral therapy, July 2017. 2017. 29341560

[38] Kerschberger B , Boulle AM , Kranzer K , Hilderbrand K , Schomaker M , Coetzee D , et al. Viral load at 3 months after initiation of antiretroviral therapy is associated with better virological and treatment outcomes than at 6 months. Int. AIDS Conf. Abstr. MOPE144 1720 July 2011. Geneva: International AIDS Society 2011.

[39] Kanters S , Vitoria M , Doherty M , Socias ME , Ford N , Forrest JI , et al. Comparative efficacy and safety of first‐line antiretroviral therapy for the treatment of HIV infection: a systematic review and network meta‐analysis. Lancet HIV. 2016;3:e510–20. 27658869 10.1016/S2352-3018(16)30091-1

[40] Elliot E , Chirwa M , Boffito M . How recent findings on the pharmacokinetics and pharmacodynamics of integrase inhibitors can inform clinical use. Curr Opin Infect Dis. 2017;30:58–73. 27798496 10.1097/QCO.0000000000000327

